# Genetic Characterization of AmpC and Extended-Spectrum Beta-Lactamase Phenotypes in *Escherichia coli* and *Salmonella* From Alberta Broiler Chickens

**DOI:** 10.3389/fcimb.2021.622195

**Published:** 2021-03-12

**Authors:** Tam Tran, Sylvia Checkley, Niamh Caffrey, Chunu Mainali, Sheryl Gow, Agnes Agunos, Karen Liljebjelke

**Affiliations:** ^1^ Department of Ecosystem and Public Health, Faculty of Veterinary Medicine, University of Calgary, Calgary, AB, Canada; ^2^ Animal Policy and Epidemiology Section, Animal Health Branch, Animal Health and Assurance Division, Alberta Agriculture and Forestry, Edmonton, AB, Canada; ^3^ Western College of Veterinary Medicine, University of Saskatchewan, Saskatoon, SK, Canada; ^4^ Center for Foodborne, Environmental and Zoonotic Infectious Diseases, Public Health Agency of Canada, Guelph, ON, Canada

**Keywords:** *Escherichia coli*, *Salmonella*, *bla*_CMY-2_, *bla*_TEM_, antimicrobial resistance genes

## Abstract

Horizontal gene transfer is an important mechanism which facilitates bacterial populations in overcoming antimicrobial treatment. In this study, a total of 120 *Escherichia coli* and 62 *Salmonella enterica* subsp. *enterica* isolates were isolated from broiler chicken farms in Alberta. Fourteen serovars were identified among *Salmonella* isolates. Thirty one percent of *E. coli* isolates (37/120) were multiclass drug resistant (resistant to ≥ 3 drug classes), while only about 16% of *Salmonella* isolates (10/62) were multiclass drug resistant. Among those, eight *E. coli* isolates had an AmpC-type phenotype, and one *Salmonella* isolate had an extended-spectrum beta-lactamase (ESBL)-type beta-lactamase phenotype. We identified both AmpC-type (*bla*_CMY-2_) and ESBL-type (*bla*_TEM_) genes in both *E. coli* and *Salmonella* isolates. Plasmids from eight of nine *E. coli* and *Salmonella* isolates were transferred to recipient strain *E. coli* J53 through conjugation. Transferable plasmids in the eight *E. coli* and *Salmonella* isolates were also transferred into a lab-made sodium azide-resistant *Salmonella* recipient through conjugation. The class 1 integrase gene, *int1*, was detected on plasmids from two *E. coli* isolates. Further investigation of class 1 integron cassette regions revealed the presence of an *aadA* gene encoding streptomycin 3’’-adenylyltransferase, an *aadA1a*/*aadA2* gene encoding aminoglycoside 3’’-O-adenyltransferase, and a putative adenylyltransferase gene. This study provides some insight into potential horizontal gene transfer events of antimicrobial resistance genes between *E. coli* and *Salmonella* in broiler chicken production.

## Introduction

For decades, antimicrobial resistance (AMR) has been a global issue of grave concern. Understanding potential mechanisms and driving forces for dissemination of genes encoding antimicrobial resistance between bacteria will help reduce the prevalence of resistant bacteria and thereby reduce risks to human and animal health. Acquisition of new resistance genes occurs frequently and naturally among bacterial communities from humans, animals and environments as outlined in the model known as ‘the epidemiology of AMR’ (Prescott, 2006). However, the mechanism of dissemination of resistance genes is not yet fully understood.

*Escherichia coli* and *Salmonella* spp. are common bacterial causes of foodborne disease in humans as well as gastrointestinal disease in animals (Folster et al., 2011; Ghodousi et al., 2015). *E. coli* is a genetically diverse species which has both commensal and pathogenic strains (Leimbach et al., 2013). *Salmonella enterica* are enteric pathogens, and are closely related to commensal *E. coli*, sharing ~85% of their genomes in common at the nucleotide level (Mcclelland and Wilson, 1998; McClelland et al., 2000). Multi-drug resistant (MDR) *E. coli* and *Salmonella* could lead to the antibiotic choices of last resort for treating multidrug-resistant Gram-negative infections. Therefore, MDR *E. coli* and *Salmonella*, especially the ones that exhibit AmpC/extended-spectrum beta-lactamase (ESBL) phenotypes, have become a major cause of clinical concern (Mohanty et al., 2010). However, these isolates are not always detected in routine susceptibility tests (Mohanty et al., 2010).

AmpC-type CMY beta-lactamase genes (*bla*_CMY_) have been found on both the chromosome and plasmids of many gram negative bacteria such as *Klebsiella* spp., *Escherichia coli*, and *Salmonella* spp. CMY-2 is reported to be the most common plasmid-carried AmpC-type CMY in both *E. coli* and *Salmonella* isolates from various global regions including Asia, North America and Europe (Guo et al., 2014). Extended spectrum beta-lactamases (ESBLs) are beta-lactamases belonging mainly to Ambler class A, which includes TEM-, SHV-, CTX-M, GES, VEB enzyme families. ESBLs also include one enzyme family, the OXA- family, belonging to class D (Cantón et al., 2012). Isolates carrying plasmid-encoded AmpC can be easily misidentified as ESBLs due to their overlapping activity against beta-lactam antimicrobials. The inability to distinguish them could have significant treatment consequences (Hanson, 2003).

Mobile genetic elements, such as plasmids or DNA transposons, are the main mechanisms facilitating horizontal genetic transfer (HGT). Plasmid-mediated *bla*_CMY-2_ has been found to be the most predominant among other acquired *ampC* genes (Mata et al., 2012). The plasmids carrying *bla*_CTX-M_ or *bla*_CMY_ beta-lactamase genes have been associated with transferable replicon types IncA/C or IncI1 (Hopkins et al., 2006; Guo et al., 2014).

Antimicrobial use in the poultry industry improves animal health, welfare and production by preventing and treating animal disease resulting in lowered mortality, but may lead to the selection of AMR organisms (Diarra and Malouin, 2014). In Canada, the preventive use of ceftiofur in broiler chicken was voluntarily eliminated by the poultry industry in May 2014; the broiler chicken antimicrobial use (AMU)-AMR surveillance component of the Canadian Integrated Program for Antimicrobial Resistance Surveillance (CIPARS) detected a significant drop in ceftiofur use at the hatcheries between 2013 and 2014 (Agunos et al., 2017). The three most frequently reported antimicrobials by producers participating in the CIPARS farm surveillance between 2013 and 2015 were bacitracin, virginiamycin and avilamycin (Agunos et al., 2017). Avilamycin was approved around May 2013 and was first reported by producers in 2014.

The isolates used in this study were a subset of those described in the 2015 CIPARS annual report (Public Health Agency of Canada, 2015). The objectives of the study were threefold: 1. To further investigate AMR phenotypes in *E. coli* and *Salmonella* isolates from broiler chickens, 2. To identify genes responsible for ESBL/AmpC phenotypes in multi-drug resistant *E. coli* and *Salmonella* isolates and 3. To conduct conjugation assays to initially assess the potential for horizontal gene transfer between *E. coli* and *Salmonella*.

## Materials and Methods

### Sampling, Bacterial Isolation, and Isolates Used in This Study

Fecal samples were taken from a single production unit on each of 30 registered premises/establishments (farms) participating in the CIPARS broiler chicken farm AMU/AMR surveillance in Alberta in 2015. Participating sentinel veterinarians were responsible for enrolling farms and collecting samples. Farms were chosen based on the veterinary practice profile and using specific inclusion and exclusion criteria. Samples were collected at pre-harvest, approximately one week prior to slaughter (broilers >30 days of age). Fecal samples consisted of 10 fecal droppings from each of the four quadrants of the chosen barn/floor, pooled to represent the chosen production unit.

For the recovery of organisms, four pooled fresh fecal samples (equivalent to at least 10 individual droppings) were collected from four quadrants of the barn. Laboratory techniques are as follows (in brief): 1) *Salmonella:* fecal samples were weighed and Buffered Peptone Water (BPW) was added (1:10) and incubated at 35°C for 24 hours. A loopful was inoculated into a Modified Semisolid Rappaport Vassiliadis (MSRV) plate and incubated at 42°C for 24 to 72 hours. 2) *E. coli:* A drop of BPW aliquot prepared from above was inoculated on MacConkey agar and incubated at 35°C for 24 hours. It is important to note that the isolates recovered here were part of the CIPARS farm surveillance component and thus no selective media were used. The intent was to harmonize with the isolation/characterization procedures outlined in the CIPARS Report (Public Health Agency of Canada, 2015).

A total number of 120 *E. coli* and 62 *Salmonella* were isolated, banked, and shipped to the University of Calgary frozen on dry ice, by the Agri Food Laboratories Section of Alberta Agriculture and Forestry (Public Health Agency of Canada, 2015; FoodNet Canada, 2017). *E. coli* J53 (KACC 16628), a recipient isolate for the conjugation experiment, was received from the Korean Agricultural Culture Collection (KACC), Agricultural Microbiology Division, National Academy of Agricultural Science. *E. coli* HB101carrying plasmid pRK600, used as a helper strain, was received from the Dong lab, University of Calgary.

### Susceptibility Tests

Minimal Inhibitory Concentrations (MICs) of various antimicrobial agents were determined using Sensititre™ (TREK Diagnostic Systems, Inc.) Gram negative plates (CMV3AGNF) designed by the National Antimicrobial Resistance Monitoring System (Public Health Agency of Canada, 2015). The same panel of antimicrobial agents was used for both *E. coli* and *Salmonella* isolates (**Tables 2**–**4**). Antimicrobial resistance assays were conducted by the National Microbiology Laboratory (NML) St. Hyacinthine, and NML Guelph (Public Health Agency of Canada, 2015).

The disc diffusion method was used to compare antimicrobial resistance profiles of isolate donors and *E. coli* recipients (Tendencia, 2004). Antibiotic discs were purchased from either BD BBL™ or Oxoid companies. The diameters of the zones of inhibition were recorded and interpreted according to Clinical and Laboratory Standards Institute (CLSI) guidelines (Clinical and Laboratory Standards Institute, 2013). For the purposes of this study, isolates displaying intermediate resistance were categorized as sensitive.

### Phenotype and Genotype Confirmation of ESBL/AmpC Genes

Two different ESBL/AmpC detection disc sets have been used to confirm ESBL/AmpC phenotypes. The first set is a combination of 4 individual discs of Cefotaxime/Cefotaxime + Clavulanic acid/Ceftazidime/Ceftazidime + Clavulanic acid, purchased from either BD BBL™ or Oxoid company. The second set is a combination of 4 individual discs of Cefpodoxime/Cefpodoxime + ESBL inhibitor/Cefpodoxime + AmpC inhibitor/Cefpodoxime + ESBL inhibitor + AmpC inhibitor, purchased from Mast Group company (D68C set).

In addition, AmpC and ESBL beta-lactamase genes were detected using PCR assays. A total of three AmpC (*bla*_CMY-2_ *, bla*_FOX_ *, bla*_ACT-1/MIR-1_) and ten ESBL (*bla*_TEM_, *bla*_SHV_ *bla*_CTX-M-1_, *bla*_CTX-M-2_, *bla*_CTX-M-8_, *bla*_CTX-M-9_, *bla*_PER-1_, *bla*_VEB_, *bla*_IBC_/*bla*_GES_, *bla*_TLA_) beta-lactamase genes were screened in AmpC/ESBL positive isolates. Primers used in the PCR assays are listed in **Table 1**. PCR products at desired sizes were purified and sent for Sanger sequencing (http://www.ucalgary.ca/dnalab/sequencing) to confirm their sequences

**Table 1:** List of primers used in this study.

Primers	PCR product	Size (bp)	Reference
CIT-A 5’-ATGCAGGAGCAGGCTATTC-3’ CIT-B 5’-TGGAGCGTTTTCTCCTGAAC-3’	*bla*_CMY-2_ FOd	689	(Mulvey et al., 2005)
fox-F 5’-TGTGGACGGCATTATCCAG-3’ fox-R 5’-AAAGCGCGTAACCGGATTG-3’	*bla*_FOX_	868	(Mulvey et al., 2005)
ent-F 5’-AGTAAAACCTTCACCTTCACCG-3’ ent-R 5’-ATGCGCCTCTTCCGCTTTC-3’	*bla*_ACT-1/MIR-1_	439	(Mulvey et al., 2005)
tem-F 5’-ATGAGTATTCAACATTTCCGT-3’ tem-R 5’-TTACCAATGCTTAATCAGTGA-3’	*bla*_TEM_	861	(Ryoo et al., 2005)
shv-F 5’-CCGGGTTATTCTTATTTGTCGCT-3’ shv-R 5’-TAGCGTTGCCAGTGCTCG-3’	*bla*_SHV_	831	(Ryoo et al., 2005)
C1-F 5’-GGACGTACAGCAAAAACTTGC-3’ C1-R 5’-CGGTTCGCTTTCACTTTTCTT-3’	*bla*_CTX-M_ (CTX-M-1 group)	624	(Ryoo et al., 2005)
C2-F 5’-CGGTGCTTAAACAGAGCGAG-3’ C2-R 5’-CCATGAATAAGCAGCTGATTGCCC-3’	*bla*_CTX-M_ (CTX-M-2 group)	891	(Ryoo et al., 2005)
C8-F 5’-ACGCTCAACACCGCGATC-3’ C8-R 5’-CGTGGGTTCTCGGGGATAA-3’	*bla*_CTX-M_ (CTX-M-8 group)	490	(Ryoo et al., 2005)
C9-F 5’-GATTGACCGTATTGGGAGTTT-3’ C9-R 5’-CGGCTGGGTAAAATAGGTCA-3’	*bla*_CTX-M_ (CTX-M-9 group)	947	(Ryoo et al., 2005)
PER-1-F 5’-GTTAATTTGGGCTTAGGGCAG-3 PER-1-R 5’-CAGCGCAATCCCCACTGT-3’	*bla*_PER-1_	855	(Ryoo et al., 2005)
VEB-F 5’-ACCAGATAGGAGTACAGACATATGA -3’ VEB-R 5’-TTCATCACCGCGATAAAGCAC-3’	*bla*_VEB_	727	(Ryoo et al., 2005)
I/G-F 5’-GTTAGACGGGCGTACAAAGATAAT-3’ I/G-R 5’-TGTCCGTGCTCAGGATGAGT-3’	*bla*_IBC/_ *bla*_GES_	903	(Ryoo et al., 2005)
TLA-F 5’-CGCGAAAATTCTGAAATGAC-3’ TLA-R 5’-AGGAAATTGTACCGAGACCCT-3’	*bla*_TLA_	992	(Ryoo et al., 2005)
ChuA.1 5’-GACGAACCA ACGGTCAGGAT-3’ ChuA.2 5’-TGCCGCCAGTACCAAAGACA-3’	*chuA*	279	(Clermont et al., 2000; Clermont et al., 2013)
YjaA.1 5’-TGAAGTGTCAGGAGACGCTG-3’ YjaA.2 5’-ATGGAGAATGCGTTCCTCAAC-3’	*yjaA*	211	(Clermont et al., 2000; Clermont et al., 2013)
TspE4C2.1 5’-GAGTAATGTCGGGGCATTCA-3’ TspE4C2.2 5’-CGCGCCAACAAAGTATTACG-3’	TspE4.C2	152	(Clermont et al., 2000; Clermont et al., 2013)
AceK.f 5′-AACGCTATTCGCCAGCTTGC-3′ ArpA1.r 5′-TCTCCCCATACCGTACGCTA-3’	*arpA*	400	(Clermont et al., 2013)
hep35 5’-TGCGGGTYAARGATBTKGATTT-3’ hep36 5’-CARCACATGCGTRTARAT-3’	*int1, int2* and *int3*	491	(White et al., 2000; White et al., 2001)
hep58 TCATGGCTTGTTATGACTGT hep59 5’-GTAGGGCTTATTATGCACGC-3’	Class1 integron casset region	variable	(White et al., 2000; White et al., 2001)
hep74 5’-CGGGATCCCGGACGGCATGC ACGATTTGTA-3’ hep51 5’-GATGCCATCGCAAGTACGAG-3’	Class2 integron cassette regions	variable	(White et al., 2001)
IncI1_F 5’-CGAAAGCCGGACGGCAGAA-3’ IncI1_R 5’-TCGTCGTTCCGCCAAGTTCGT-3’	RNAI	139	(Carattoli et al., 2005)
IncA/C_F 5’-GAGAACCAAAGACAAAGACCTGGA-3’ IncA/C_R 5’-ACGACAAACCTGAATTGCCTCCTT-3’	repA	465	(Carattoli et al., 2005)

Multi-drug resistant (MDR) *E. coli* and *Salmonella* isolates that were resistant to at least three drug classes and were confirmed to exhibit ESBL/AmpC phenotypes were selected for additional experiments. Seven drug classes were used in this study: beta-lactams (AMC = Amoxycillin + Clavulanic acid, AMP = Ampicillin, FOX = Cefoxitin, CRO = Ceftriaxone, TIO = Ceftiofur), aminoglycosides (GEN = Gentamicin, STR = Streptomycin), quinolones (NAL = Nalidixic acid, CIP = Ciprofloxacin), sulfonamides (SSS = Sulfisoxazole, SXT = Trimethoprim sulfamethoxazole), macrolides (AZM = Azithromycin), phenicols (CHL = Chloramphenicol), tetracyclines (TET =Tetracycline).

### Plasmid Characterization/Replicon Typing

Plasmid miniprep was performed using an alkaline lysis method (Birnboim and Doly, 1984). The plasmid size was evaluated using agarose gel electrophoresis.

Replicon typing was performed using PCR assay as described previously, with primers listed in **Table 1** (Carattoli et al., 2005).

### *E. coli* Phylogroups/*Salmonella* Serovars

MDR *E. coli* isolates exhibiting ESBL/AmpC phenotypes were assigned into one of four main phylogenetic groups by using a simplified two-step triplex polymerase reaction (Clermont et al., 2000). The results were confirmed using a quadruplex PCR assay which enabled us to classify isolates into a broader range of *E. coli* phylogroups as well as distinguish them from the cryptic clades II to V (Clermont et al., 2013).

An assay to classify *Salmonella* serovars was performed by the PHAC serotyping laboratory as described previously (Public Health Agency of Canada, 2015).

### Detection of Integrons/Integrases

To further study other mobile genetic elements, *int1*, *int2* and *int3* genes encoding integrase of class 1, 2 and 3 integrons, respectively, were investigated by PCR assays using primers listed in **Table 1** as described previously (White et al., 2001). Primers for amplifying the class 1 and class 2 integron cassette regions were used to detect the presence of resistance gene cassettes (**Table 1**) (White et al., 2001). PCR products were purified and sent for Sanger sequencing (http://www.ucalgary.ca/dnalab/sequencing) to confirm their sequences.

### Conjugation Experiment

Conjugation experiments were conducted using MDR isolates of interest as donors and *E. coli* J53 as the recipient with or without the presence of helper strain HB101/pRK600. *E. coli* J53 (F- *met pro* Azi^r^ Amp^s^), an *E. coli* K-12 derivative strain, is resistant to sodium azide but sensitive to beta-lactams (Yi et al., 2012). This strain is also sensitive to other drugs (e.g. tetracycline, chloramphenicol, gentamicin, streptomycin) (Lei et al., 2019). Recipient and donor strains were inoculated into LB broth and cultured overnight at 37°C. The next day, cells were harvested, washed with saline, and mixed together in a ratio of 1:1, and spotted on to LB plates. They were also spotted individually on LB plates as controls. After overnight incubation at 37°C, mating spots were washed and resuspended in saline; and different dilutions were plated on LB media containing sodium azide (0.2 gL^-1^) and ampicillin (100 µgml^-1^) to select transconjugants. Control spots were transferred to the same selective media to make sure that no growth was observed. Conjugation frequency was calculated by taking the ratio of the number of colonies counted on selective plates (LB supplemented with sodium azide (0.2 gL^-1^) + ampicillin (100 µgml^-1^)) for transconjugants over the number of colonies on selective plates (LB supplemented with sodium azide (0.2 gL^-1^)) for recipients. If there were no transconjugants obtained, a helper strain (HB101/pRK600) was added into the mating mix in the proportion of 1:1:0.5 (donor: recipient: helper strain) and spotted on LB plates as described. If there was no growth on plates selected for recipients, trypsin was added to the media to reverse the effect of colicin produced by the donors, and recover the recipients (Nomura and Nakamura, 1962; Dankert et al., 1980).

*Salmonella* isolate 112.2 was screened for spontaneously mutated colonies resistant to sodium azide (Azi^R^) by plating on LB supplemented with sodium azide (0.2 gL-1). Then this Azi^R^ *Salmonella* was used as a recipient in conjugation with MDR isolates of interest as donors. Conjugation protocol was performed as described above.

### Data Visualization Tools

Data visualization in this study was performed using following programs: Microsoft Excel 2013, R programming (R version 3.4.1).

## Results

### Sampling, Isolation and Identification of Bacterial Strains

Four *E. coli* isolates were obtained from each farm, resulting in 120 *E. coli* isolates from 30 farms with a recovery rate of 100% (30/30). There were 23 of 30 farms *Salmonella* positive, with between one and four isolates identified per farm. Hence the recovery rate for *Salmonella* was about 77% (23/30). There were 14 different serovars identified among 62 *Salmonella* isolates (**Table 2**). The three most prevalent *Salmonella* serovars in our study were Enteritidis, Hadar, and Thompson.

**Table 2:** AMR patterns in *Salmonella* isolates along with their serotypes.

Salmonella serotype	Resistance pattern																	Ratio^a^	
	beta-lactam					Macrolide		Phenicol	Quinolone			Aminoglycoside		Sulfonamide			Tetracycline		
	AMC	AMP	CRO	FOX	TIO	AZM		CHL	CIP	NAL		GEN	STR	SSS	SXT		TET		
Enteritidis																		10/10	
Braenderup																		1/1	
Hadar																			
Pattern 1		R											R				R	6/10	
Pattern 2		R															R	1/10	
Pattern 3													R				R	3/10	
Hartford																		1/1	
Heidelberg		R	R										R				R	1/1	
Infantis																		9/9	
Kentucky													R				R	1/1	
Mbandaka																			
Pattern 1													R	R			R	1/4	
Pattern 2																	R	3/4	
Schwarzengrund																		2/2	
Senftenberg																		5/5	
Thompson																		10/10	
Typhimurium																		4/4	
Worthington													R	R			R	2/2	
I 6,7:k:-																		2/2	
Total																		62/62	

There were seven drug classes tested: 1. Beta-lactam (AMC, Amoxycillin + Clavulanic acid; AMP, Ampicillin; FOX, Cefoxitin; CRO, Ceftriaxone; TIO, Ceftiofur), 2. Aminoglycosides (GEN, Gentamicin; STR, Streptomycin), 3. Quinolones (NAL, Nalidixic acid; CIP, Ciprofloxacin), 4. Sulfonamides (SSS, Sulfisoxazole; SXT, Trimethoprim sulfamethoxazole), 5. Macrolides (AZM, Azithromycin), 6. Phenicols (CHL, Chloramphenicol), 7. Tetracyclines (TET, Tetracycline). Drugs were grouped in classes by clear/grey areas.

R, Resistant.

^a^Ratio is defined as the number of isolates of the same resistance pattern over the total number of isolates.

All the antimicrobials belonging to the same drug class were placed next to each other and separated from those in other drug classes by shading.

### Antimicrobial Susceptibility Testing

Antimicrobial resistance in *E. coli* and *Salmonella* isolates is described as follows. Isolates that were resistant to three or more antimicrobial classes were considered MDR. Thirty-one percentage of *E. coli* (37/120) were MDR and 16% of *Salmonella* (10/62) were MDR. About 4% of *E. coli* (5/120) were resistant to five antimicrobial classes, while none of *Salmonella* were resistant to five classes.

**Figure 1:**
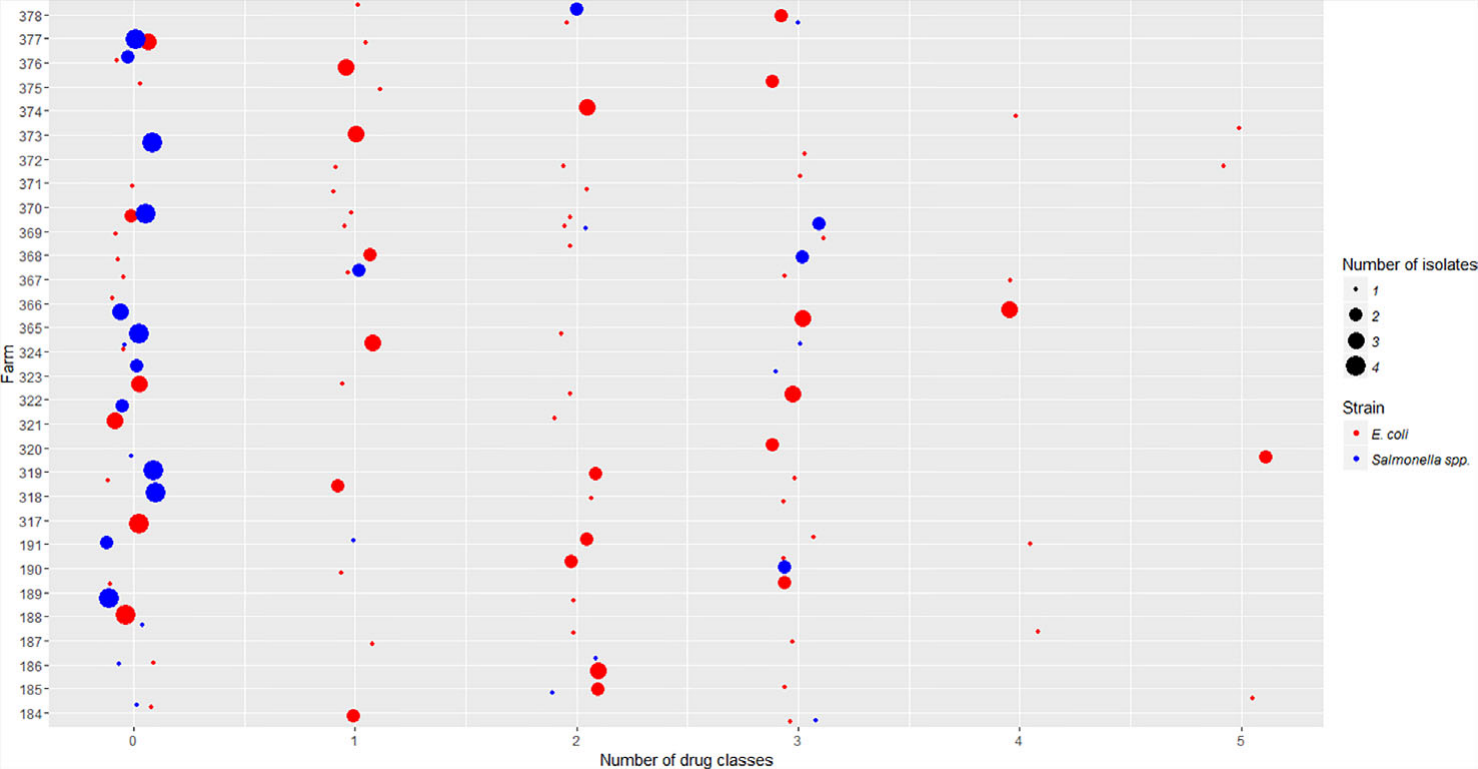
*Salmonella* isolates resistant to differing number of drug classes were compared to *E. coli* isolates across participating farms. The x-axis is the number of drug classes to which isolates showed resistance. The y-axis is the code of individual farms participating in this project. The size of each dot represents the number of isolates obtained in each farm. The color represents whether the isolates are *E. coli* or *Salmonella* strains.

The majority of *Salmonella* isolates were resistant to streptomycin and tetracycline (**Table 2**). There were 8 *Salmonella* serotypes that were sensitive to all tested antimicrobials (Enteritidis, Typhimurium, Braenderup, Hartford, Infantis, Schwarzengrund, Senftenberg, Thompson). In addition to streptomycin and tetracycline, the majority of MDR *E. coli* showed resistance to sulfisoxazole (**Table 3**). Among *E. coli* that were phylo-typed, those belonging to groups D or E had diverse AMR patterns (**Table 4**). Overall, *E. coli* isolates showed more diversity in resistance phenotype between farms than did *Salmonella* (**Figure 1**).

**Table 3:** AMR patterns in all MDR *E.coli* isolates (≥3 drug classes).

Resistance to # classes	Resistance pattern																	Ratio^a^
	beta-lactam					Macrolide		Phenicol	Quinolone			Aminoglycoside		Sulfonamide			Tetracycline	
	AMC	AMP	CRO	FOX	TIO	AZM		CHL	CIP	NAL		GEN	STR	SSS	SXT		TET	
5	R	R	R	R	R			R					R	R			R	3/5
	R	R	R	R	R			R				R	R	R			R	2/5
4	R	R	R	R	R							R	R	R			R	1/7
		R										R		R			R	4/7
		R											R	R			R	2/7
3	R	R	R	R	R								R				R	1/25
	R	R	R	R	R					R			R					1/25
	R	R											R				R	1/25
		R										R	R				R	1/25
		R										R	R	R	R			1/25
		R											R	R	R			1/25
		R											R				R	1/25
								R						R			R	1/25
												R	R	R			R	3/25
												R		R			R	7/25
													R	R	R		R	1/25
													R	R			R	6/25
Total																		37/37

There were seven drug classes: 1. Beta-lactam (AMC, Amoxycillin + Clavulanic acid; AMP, Ampicillin; FOX, Cefoxitin; CRO, Ceftriaxone, TIO, Ceftiofur), 2. Aminoglycosides (GEN, Gentamicin; STR, Streptomycin), 3. Quinolones (NAL, Nalidixic acid; CIP, Ciprofloxacin), 4. Sulfonamides (SSS, Sulfisoxazole; SXT, Trimethoprim sulfamethoxazole), 5. Macrolides (AZM, Azithromycin), 6. Phenicols (CHL, Chloramphenicol), 7. Tetracyclines (TET, Tetracycline). Drugs were grouped in classes by clear/grey areas. Antimicrobials belonging to the same drug class were placed next to each other and separated from those in other drug classes by shading. ^a^Ratio is defined as the number of isolates of the same resistance pattern over the total number of isolates.

**Table 4:** AMR patterns in eight MDR *E. coli* strains carrying plasmids and their phylogroups.

*E. coli* phylogroup	Resistance pattern																	Number of isolates	Ratio^a^
	beta-lactam					Macrolide		Phenicol	Quinolone			Aminoglycoside		Sulfonamide			Tetracycline		
	AMC	AMP	CRO	FOX	TIO	AZM		CHL	CIP	NAL		GEN	STR	SSS	SXT		TET		
A or C	R	R	R	R	R			R					R	R			R	3	3/3
D or E																			
57.1	R	R	R	R	R								R				R	1	1/4
58.1	R	R	R	R	R			R				R	R	R			R	1	1/4
61.1	R	R	R	R	R							R	R	R			R	1	1/4
113.1	R	R	R	R	R					R			R					1	1/4
B1	R	R	R	R	R			R				R	R	R			R	1	1/1
Total																		8	8/8

There were seven drug classes: 1. Beta-lactam (AMC, Amoxycillin + Clavulanic acid; AMP, Ampicillin; FOX, Cefoxitin, CRO=Ceftriaxone; TIO, Ceftiofur), 2. Aminoglycosides (GEN, Gentamicin; STR, Streptomycin), 3. Quinolones (NAL, Nalidixic acid; CIP, Ciprofloxacin), 4. Sulfonamides (SSS, Sulfisoxazole; SXT, Trimethoprim sulfamethoxazole), 5. Macrolides (AZM, Azithromycin), 6. Phenicols (CHL, Chloramphenicol), 7. Tetracyclines (TET, Tetracycline). Drugs were grouped in classes by clear/grey areas.

R, Resistant.

^a^Ratio is defined as the number of isolates of the same resistance pattern over the total number of isolates. All the antimicrobials belonging to the same drug class were placed next to each other and separated from those in other drug classes by shading.

Among MDR isolates, 19 *E. coli* and 10 *Salmonella* isolates with resistance to the beta-lactam class of antimicrobials and at least two other drug classes, were selected for further study. These isolates came from 19 farms.

### ESBL/AmpC Phenotypes and Genotypes

Eight out of 19 MDR *E. coli* isolates were resistant to both penicillin and cephalosporin beta-lactam sub-classes and were confirmed as AmpC phenotype (**Table 5**). A unique *Salmonella* was resistant to both penicillin and cephalosporin sub-classes and was potentially an ESBL phenotype; in the presence of an ESBL inhibitor (clavulanic acid) the isolate showed sensitivity to penicillin (specifically amoxicillin). The ESBL phenotype was subsequently confirmed by disc diffusion method.

**Table 5:** Characteristics of MDR *E. coli* and *Salmonella* isolates showing ESBL/AmpC phenotypes.

Strain	IsolateNumber	Phylogroup (*E.coli*)/Serotype (*Salmonella*)	Plasmid type	ESBL/AmpC phenotype	*bla*_CMY-2_ gene	*bla*_TEM-116_ gene	Integron/Integrase
*E. coli*	11.1 12.1 57.1 58.1 61.1 82.1 89.1 113.1	A^a^/A or C^b^ A^a^/A or C^b^ D^a^/D or E^b^ D^a^/D or E^b^ D^a^/D or E^b^ B1^a,b^ A^a^/A or C^b^ D^a^/D or E^b^	A/C, I1 A/C, I1 A/C, I1 A/C, I1 A/C, I1 A/C, I1 A/C, I1 A/C, I1	AmpC AmpC AmpC AmpC AmpC AmpC AmpC AmpC	+ + + + + + + +	+ + + + + + + +	*aadA, aadA1a/A2* *int1*, *aadA* *int1, putative aadA1*
*Salmonella*	119.2	Heidelberg	A/C, I1	ESBL	+	+	

^a,b^ E. coli isolates were assigned into different phylogenetic group using both triplex and quadruplex PCR methods as described in previous studies, respectively (Clermont et al., 2000; 2013).

Plasmids extracted from nine isolates which either showed the AmpC or ESBL phenotype were used as templates in PCR reactions to detect a variety of AmpC and ESBL genes. The *bla*_CMY-2_ gene, an AmpC-type gene, and the *bla*_TEM_ **gene, an ESBL-type gene, were identified on plasmids from all nine *E. coli* and *Salmonella* isolates. The sequence of the *bla*_TEM_ gene identified in this study shared 100% identity with the sequence of the *bla*_TEM-116_ gene found in *E. coli* strain MRC3 (accession no. KJ923009.1)

### Plasmid Characterization

Nine isolates were found to carry IncI1 and IncA/C-type replicon plasmids (**Table 5**). The plasmids varied in size from approximately 7kb to larger than 20kb. The plasmid in one *Salmonella* isolate has a size larger than 20 kb with I1 and A/C-type replicon. Two *E. coli* isolates, 58.1 and 61.1, carried small plasmids (<10kb), while the rest carried larger ones (≥20 kb). All plasmids in *E. coli* isolates had IncI1 and IncA/C-type replicons.

### *E. coli* Phylogroups/*Salmonella* Serovars of ESBL/AmpC-Positive Isolates

Both PCR methods confirmed that none of the *E. coli* isolates belonged to the group B2 (a group with high potential for pathogenicity) (**Tables 4** and **5**). Phylogroups of *E. coli* identified in this study were A, B_1_ and D (triplex PCR) or A, B_1_, C, D and E (quadruplex PCR). One MDR *Salmonella* isolate exhibiting the ESBL phenotype was identified as Heidelberg serovar.

### Detection of Integrons/Integrases

Using DNA from plasmid extraction as a template in PCR reactions, the class 1 integrase gene *int1* was detected in two *E. coli* isolates that were from 2 different farms (**Table 5**). The class 1 integron cassette region was also detected in three isolates by PCR (**Table 5**). The PCR products were sequenced, and the results were confirmed by blasting the sequence against the NCBI database. The sequence matched the aminoglycoside resistance genes, *aadA* encoding streptomycin 3’’-adenylyltransferase, *aadA1a*/*aadA2* encoding aminoglycoside 3’’-O-adenyltransferase. The class 1 integron cassettes were also amplified from plasmid extraction from *E. coli* isolate 82.1. The results were confirmed by blasting the sequence against the NCBI database. The sequences matched the sequence of a putative adenylyltransferase found on a plasmid isolated from the *Salmonella* Heidelberg strain N418 (Accession no. CP009409).

### Transfer of Plasmids Carrying Resistance Genes by Conjugation

Plasmids were mobilized from all, but one *E. coli* isolate 89.1, to an *E. coli* recipient J53 (**Table 6**). No growth was observed on selective plates of either the transconjugants or recipients when attempting to conjugate *E. coli* isolate 61.1. However, the conjugation experiment of this isolate was successful when the media were supplemented with trypsin to reverse the effect of colicin produced by the donors. Additionally, the *E. coli* isolate 113.1 required the presence of the helper *E. coli* strain HB101 carrying helper plasmid pRK600 to enable movement of the plasmid to recipients. All transconjugants were resistant to ampicillin which was used as a selective marker in conjugation experiments. Interestingly, their resistance phenotypes to other drugs, which were presented as “transferable AMR” in **Table 6**, were distinctly different, except for recipients that were conjugated with either donor 12.1 or 113.1. Although transconjugants obtained from conjugation assays with either donor 12.1 or 113.1 were resistant to the same drugs including amoxicillin, cefoxitin, ceftriaxone and ceftiofur, donor 12.1 produced *E. coli* transconjugants more efficiently (higher conjugation frequency) and did not require the presence of the helper strain (**Table 6**).

**Table 6:** Conjugation frequency and AMR profile of transconjugants compared to donors (tested isolates) using *E. coli* J53 as a recipient.

Donor Strain	IsolateNumber	Conjugation frequency	Transferable AMR	Non-transferable AMR
*E. coli*	11.1 12.1 57.1 58.1 61.1^b^ 82.1 89.1 113.1^c^	4 X 10^-5^ 13 X 10^-3^ 14 X 10^-5^ 3 X 10^-1^ 3 X 10^-4^ 5 X 10^-2^ Non-transferable 3 X 10^-4^	AML, FOX, STR, TIO, CHL^a^, CRO, TET AML, FOX, TIO, CRO AML, STR, TET AML, FOX, GEN, STR, TIO, CHL, CRO, TET AML, FOX AML, FOX, GEN, STR, TIO, CHL^a^, CRO N/A AML, FOX, CRO, TIO	NAL NAL, CHL, TET FOX, TIO, CRO - TIO, CRO, GEN, STR, TET, TIO TET AML, FOX, STR, TIO, CHL, CRO, TET NAL, STR
*Salmonella* (Heidelberg)	119.2	9 X 10^-2^	AML, TIO^a^, CRO^a^, TET^a^	STR

Antimicrobial abbreviation: AML, Amoxicillin; FOX, Cefoxitin; CRO, Ceftriaxone; GEN, Gentamicin; NAL, Nalidixic acid; CHL, Chloramphenicol; TET, Tetracycline; TIO, Ceftiofur; STR, Streptomycin.

a Results were interpreted as intermediate (between resistant and sensitive) in recipient E. coli strains while donor strains (original isolates) were resistant to these antimicrobial agents.

b Donor strains produced colicin which is lethal to recipient strains. Trypsin was added into the media to rescue recipient strains.

c Helper strains were required to help transfer plasmid from donor strains to recipient strains.

When using a lab-engineered sodium azide-resistant *Salmonella* as a recipient, we observed that plasmids from eight *E. coli* isolates with the exception of the isolate 89.1 mentioned above, were able to move to *Salmonella* with variable conjugation frequency (**Table 7**).

**Table 7:** Conjugation frequency between donors (tested isolates) and a recipient strain (sodium azide-resistant *Salmonella*).

Strain	Isolate ID Number	Conjugation frequency	Isolate ID Number	Conjugation frequency
*E. coli*	11.1	4 X 10^-9^	61.1	5 X 10^-4^
	12.1	1 X 10^-5^	82.1	2 X 10^-2^
	57.1	3 X 10^-7^	89.1	Non-transferable
	58.1	7 X 10^-7^	113.1	2 X 10^-4^
*Salmonella* (Heidelberg)	119.2	6 X 10^-2^		

### Farm Characteristics for Nine Isolates From Which Plasmids Were Mobilized

Seven ‘conventional’ (i.e., antimicrobials were used to some extent in all flocks) farms under the veterinary care of one practice were represented by the nine ESBL/AmpC phenotyped isolates in this study. All of the farms, excluding the farm providing isolate 89.1, received their chicks from the same hatchery. All birds were Ross 308 strain. The most frequently used antimicrobials were bacitracin and salinomycin administered *via* feed (n = 5), followed by the combination of penicillin and streptomycin administered *via* water (n = 3). In addition, the feed were also reportedly medicated with the following antimicrobials avilamycin (n = 1), tylosin (n = 2), and coccidiostats such as decoquinate (n = 1), monensin (n = 1), the ionophore-chemical coccidiostat combination, narasin and nicarbazin (n = 2).

The number of chicks sampled per flock ranged from 14,790 to 55,000 within a single production unit. Age on the day of sampling ranged from 30 to 35 days old with an average weight ranging from 1.7 kg to 2.2 kg. The recorded floor space in the barns ranged from 8000 ft^2^ to 30550 ft^2^ and stocking density ranged from 0.54 to 0.67 ft^2^ per bird. Reported percentage of mortality ranged from 2.47% to 7.19% of the birds placed within the barn.

Hydrogen peroxide was used on three of the farms for cleaning of water lines between flocks. Five of the farms also used chlorine for treatment of their water lines during the production cycle. Footbaths (n = 3), dedicated farm clothes (n = 4) and gloves (n =2) were methods of farm biosecurity utilized. Manure was stored onsite in the vicinity of the barn on three farms. The most frequently reported method of cleaning the barns after each production cycle was washing only (n = 6) and chlorine products were used for disinfection on four farms.

## Discussion

The three most prevalent *Salmonella* serovars in our study were Enteritidis, Hadar, and Thompson. Serovars Typhimurium and Heidelberg were also identified. According to the National Enteric Surveillance Program (NESP) 2013 Annual Report, the three most commonly reported serovars from human cases in Canada, which has remained unchanged since 2008, were Enteritidis, Heidelberg and Typhimurium (Government of Canada, 2015). Serovar Enteritidis is known as one of the most common *Salmonella* serovars found in broiler chickens, and the second most prevalent cause of *Salmonella* infection in humans after the serovar Typhimurium (Suzuki, 1994; Porwollik et al., 2005; Trampel et al., 2014). Serovars Hadar, Heidelberg, Mbandaka and Worthington were MDR. Of the 14 serovars in our study, the *Salmonella* serovar Heidelberg was the only MDR isolate with an ESBL phenotype. This is a of concern because in 2013-2014, a national outbreak of MDR *Salmonella* Heidelberg infections in the United States resulted in 200 hospitalized cases of 528 total cases (38%) (Gieraltowski et al., 2016). This outbreak was linked to chicken products from a single poultry company. In our study, six of the seven farms had chicks sourced from the same hatchery. There is potential for widespread dissemination of virulent bacteria over a wide geographical region if such strains are present among eggs or chicks at the hatchery level. However, there was no evidence of this in our study.

Urinary tract infections are one of the most common bacterial infections reported in primary care and the emergency department in Canada (Sanyal et al., 2019). Phylogroup B2 has been considered to be the most extra-intestinal virulent group (Clermont et al., 2000), and shown to have a strong association with the uropathogenic subpathotype (Hutton et al., 2018). To determine whether the MDR *E. coli* isolates that exhibited AmpC or ESBL phenotypes belonged to phylogroup B2, both triplex and quadruplex PCR assays were performed. Both assays confirmed that these isolates did not belong to group B2, therefore less likely caused urinary tract infections.

Both *bla*_CMY-2_ and *bla*_TEM_ genes may be present on plasmids isolated from AmpC/ESBL positive MDR *E. coli* and *Salmonella* isolates. The *bla*_CMY-2_ and *bla*_TEM_ genes had identical sequences in both *E. coli* and *Salmonella* isolates in this study. A previous study showed the evidence for the transfer of *bla*_CMY-2_- carrying plasmids between *E. coli* and *Salmonella* isolates (Winokur et al., 2001). Interestingly, *E. coli* isolates had the AmpC beta-lactamase phenotype while *Salmonella* had the ESBL beta-lactamase phenotype. Even though both species carried both *bla*_CMY-2_ and *bla*_TEM_ genes, the phenotype differences suggest differential expression of these genes in these *E. coli* and *Salmonella* isolates.

The *bla*_CMY-2_ gene is the most common AmpC-type gene found in both *E. coli* and *Salmonella* from various sources: food, animals, and hospitals in multiple countries (Mulvey et al., 2005; Hiki et al., 2013; Cejas et al., 2014; Guo et al., 2014; Ghodousi et al., 2015). This gene has been hypothesized to have originated on the chromosome of *E. coli* and it could be induced with beta-lactams in some *Enterobacteriaceae* such as *Enterobacter cloacae, Citrobacter freundii, Serratia marcescens*, and *Pseudomonas aeruginosa* (Sanders, 1987; Philippon et al., 2002). Unlike these bacteria, *E. coli* and *Salmonella* lack systems to produce inducible AmpC enzymes. Mutations in the *ampC* promoter have increased the resistance to oxyimino-cephalosporins in *E. coli* (Caroff et al., 1999).

Plasmids are considered to be facilitators for disseminating beta-lactamase genes between various species such as *P. mirabilis*, *Achromobacter*, *Salmonella* and *E. coli* (Bobrowski et al., 1976; Levesque et al., 1982; Knothe et al., 1983; Bauernfeind et al., 1989). Molecular characterization of MDR plasmids is essential, yet complicated, because these plasmids are very diverse and promiscuous. The relatedness of plasmids can be analyzed using a PCR-based replicon typing method or whole genome sequence analysis (Carattoli et al., 2005). In previous studies, *bla*_CMY-2_-carrying plasmids found in either *E. coli* or *S. enterica* were most likely to belong to replicons I1 and A/C (Carattoli, 2009). Our results are also in accordance with these findings. *bla*_TEM_ genes have been reported to be located on plasmids of various replicon types such as A/C, I1, K, ColE, H12, etc. (Carattoli, 2009). In our study, *bla*_TEM_ gene was found on plasmids of replicons A/C or I1 in one *Salmonella* isolate and eight *E. coli* isolates. Previously, it was shown that plasmids encoding ESBL/AmpC genes in *E. coli* were highly promiscuous, resulting in the possibility of HGT between *E. coli* and related *Enterobacteriaceae* strains (Ewers et al., 2012).

In addition to plasmids, other mobile genetic elements (e.g. integrons containing gene cassettes and integrase) are also facilitating the spread of AMR genes (White et al., 2001). It was shown in multiple independent reports that there was an occurrence of integrons especially class 1 integrons and AMR genes (Leverstein-van Hall et al., 2002; Marashi et al., 2012; Di Cesare et al., 2016). More specifically, a significant association was found between integrons and resistance to certain antimicrobials including gentamicin, kanamycin, streptomycin, tobramycin, sulfafurazole, trimethoprim, ampicillin, chloramphenicol, and tetracycline (White et al., 2001). In our study, the integrase gene *int1* was detected in two out of nine AmpC/ESBL-producing isolates. Using specific primers to amplify class 1 integron cassette regions revealed the presence of the aminoglycoside resistance genes *aadA* encoding streptomycin 3’’-adenylyltransferase, *aadA1a*/*aadA2* encoding aminoglycoside 3’’-O-adenyltransferase, and a putative adenylyltransferase gene. *E. coli* isolate 11.1 carried a gene cassette in its variable region but did not carry the *int1* gene of the classical class 1 integron. This might be due to the disruption caused by IS*26* which was reported in a previous study (Dawes et al., 2010).

Conjugation *in vitro* showed that most of AmpC/ESBL positive MDR isolates carried transferable plasmids that can disseminate AMR phenotypes. The conjugation experiment on isolate 61.1 required the addition of trypsin into the media. Previous studies have shown that treatment of cells with trypsin reversed the inhibition activity caused by colicin (Nomura and Nakamura, 1962; Dankert et al., 1980). The isolate 61.1 likely harbored a colicin-producing plasmid which prevented conjugal transfer of the R-plasmid; hence, this would likely prevent conjugal transfer in the natural microbial community as well. It is interesting to note that in this study all ESBL/AmpC-producing MDR isolates, except for *E. coli* isolate 89.1, were from farms receiving their chicks from the same hatchery; and *Salmonella* serovar Heidelberg was one of them. In addition, we were able to transfer plasmids between strains: from *E. coli* to *E. coli*, from *E. coli* to *Salmonella*, from *Salmonella* to *E. coli*, and from *Salmonella* to *Salmonella*. Plasmids were transferable through conjugation from *E. coli* to *E. coli* or *Salmonella* to *E. coli* at higher frequencies compared to plasmids from *E. coli* to *Salmonella* or *Salmonella* to *Salmonella*.

In conclusion, this study investigated antimicrobial resistance phenotypes of *Escherichia coli* and *Salmonella* isolates from 30 broiler farms, which were obtained through the Canadian Integrated Program for Antimicrobial Resistance Surveillance. The study subsequently identified MDR isolates of *E. coli* and *Salmonella enterica* with ESBL/AmpC phenotypes and examined the sequences of the ESBL/AmpC genes in these isolates. In addition, plasmids from these MDR isolates were isolated and shown to carry the identical replicon type. We also performed conjugation assays between *E. coli* and *Salmonella* isolates to initially assess the potential for HGT. Overall, results suggested there are MDR bacteria in broiler chicken environments with characteristics that could potentially allow them to flourish in the broiler environment, and the possibility of natural HGT by conjugation between *E. coli* and *Salmonella* may readily occur in the broiler chicken house environment.

## Data Availability Statement

The raw data supporting the conclusions of this article will be made available by the authors, without undue reservation.

## Author Contributions

SC, KL, SG, and CM conceptualized the research idea and obtained research funding from AAF (PI: SC). SG and AA developed the CIPARS AMU-AMR farm surveillance framework, farm surveillance tools (questionnaire) and protocols, and validated the recovery and AMR datasets. Bacterial isolation and initial antimicrobial susceptibility testing were performed by RC. TT and KL were responsible for experimental design. TT conducted research and laboratory analysis. NC conducted statistical analysis. TT and NC designed and drafted the manuscript. All authors contributed to the article and approved the submitted version.

## Funding

This study was funded by Alberta Agriculture and Forestry (AAF) [grant number 2015R025R] with significant in a kind support from PHAC and the AAF, Agri-Food Laboratories.

## Conflict of Interest

The authors declare that the research was conducted in the absence of any commercial or financial relationships that could be construed as a potential conflict of interest.
